# Effectiveness and safety of bee venom pharmacopuncture for rheumatoid arthritis-related pain and disease activity: a systematic review and meta-analysis

**DOI:** 10.3389/fmed.2026.1954842

**Published:** 2026-10-14

**Authors:** Jinyeong Hong, Won-Suk Sung, Eun-Jung Kim, Byung-Kwan Seo, Joo-Hee Kim

**Affiliations:** 1 Department of Acupuncture and Moxibustion Medicine, College of Korean Medicine, Sangji University, Wonju-si, Republic of Korea; 2 Department of Acupuncture and Moxibustion, Dongguk University Bundang Oriental Hospital, Seongnam-si, Gyeonggi-do, Republic of Korea; 3 Department of Acupuncture and Moxibustion Medicine, College of Korean Medicine, Kyung Hee University Hospital at Gangdong, Kyung Hee University, Seoul, Republic of Korea; 4 Research Institute of Korean Medicine, Sangji University, Wonju-si, Republic of Korea

**Keywords:** bee venom, disease activity, meta-analysis, pain, pharmacopuncture, rheumatoid arthritis, systematic review

## Abstract

**Background:**

Rheumatoid arthritis (RA) is a chronic autoimmune disease characterized by joint pain, inflammation, and progressive joint destruction. Bee venom (BV) pharmacopuncture has been used in East Asia for RA, but its clinical effectiveness and safety remains uncertain. We evaluated BV pharmacopuncture for pain, disease activity, treatment response, and adverse events (AEs) in patients with RA.

**Methods:**

We systematically searched PubMed, the Cochrane Library, Embase, SciELO, CNKI, CiNii, KISS, and other regional databases for randomized controlled trials (RCTs). Risk of bias was assessed using RoB 2, meta-analyses were conducted using RevMan 5.4, and the certainty of evidence was evaluated using GRADE.

**Results:**

Seventeen RCTs involving 1,304 patients with RA were included, and 14 contributed to meta-analysis. Compared with Western medicine, BV significantly improved pain-related outcomes as monotherapy (standardized mean difference [SMD] −0.99, 95% confidence interval [CI] −1.37 to −0.60; I^2^ = 20%) and add-on therapy (SMD −1.14, 95% CI −2.11 to −0.17; I^2^ = 93%). Only one study evaluated disease activity using the Disease Activity Score in 28 joints based on the erythrocyte sedimentation rate and reported a significant benefit with BV. Although most other disease activity–related outcomes favored BV, the overall effect remained uncertain because the evidence was limited and heterogeneous. BV improved the response rate, defined as a ≥ 30% improvement, as add-on therapy (risk ratio [RR] 1.18, 95% CI 1.03 to 1.36; I^2^ = 9%), but not in direct comparisons with Western medicine (RR 1.07, 95% CI 1.00 to 1.16; I^2^ = 0%; *p* = 0.06). The risk of AEs was relatively low with BV monotherapy (risk difference [RD] −0.17, 95% CI −0.34 to −0.01; I^2^ = 37%) and add-on comparisons (RD = −0.16, 95% CI −0.26 to −0.06; I^2^ = 0%). No serious BV-related AEs, including anaphylaxis, were reported.

**Conclusions:**

BV pharmacopuncture may improve pain and treatment response in RA without increasing the risk of AEs. However, these findings are supported by low certainty of evidence, and the effect on disease activity remains uncertain. Further high-quality RCTs with standardized treatment protocols and validated outcome measures are warranted to establish its clinical efficacy and safety.

**Systematic Review Registration:**

https://www.crd.york.ac.uk/PROSPERO/view/CRD42021238058, PROSPERO CRD42021238058.

## 1 Introduction

Rheumatoid arthritis (RA) is a chronic inflammatory autoimmune disease characterized by synovitis, which leads to joint pain, swelling, morning stiffness, cartilage destruction, and progressive joint deformity (1). Although its precise etiology remains unclear, RA is generally considered to result from the interaction of genetic and environmental factors, leading to the breakdown of immune tolerance and subsequent autoimmune responses (2, 3). Persistent synovial inflammation eventually causes progressive cartilage destruction and bone erosion, resulting in joint deformity and disability (4, 5). Furthermore, prolonged nociceptive input from inflamed joints may induce central sensitization, contributing to persistent pain even after inflammation has been adequately controlled (6, 7). Consequently, joint damage and persistent pain substantially impair physical function and reduce health-related quality of life (8, 9).

The primary goal of RA treatment is to reduce disease activity to prevent irreversible joint damage and deformity while minimizing the risk of extra-articular involvement. Current pharmacological management of RA is primarily based on disease-modifying antirheumatic drugs (DMARDs) (10, 11). Methotrexate (MTX), a conventional synthetic DMARD (csDMARD), is the recommended first-line therapy (12). However, a substantial proportion of patients fail to achieve adequate disease control with MTX monotherapy, necessitating treatment escalation or combination therapy with other agents, such as leflunomide (LEF), sulfasalazine (SSZ), and biologic and targeted synthetic DMARDs (13, 14). These agents are also associated with a range of adverse events (AEs), including hepatotoxicity, rash, alopecia, and gastrointestinal complications such as gastric ulcers and diarrhea (15–17). Moreover, treatment discontinuation remains common in clinical practice because of inadequate efficacy and treatment-related AEs (18, 19). Consequently, there has been growing interest in complementary therapeutic approaches as adjuncts to conventional pharmacological therapy for improving symptom control and reducing the overall treatment burden (20, 21).

Bee venom (BV), a natural substance derived from honeybees (*Apis cerana and Apis mellifera*), is a complex mixture of bioactive peptides, enzymes, and biogenic amines. Through the combined actions of its bioactive components, BV exerts anti-inflammatory, analgesic, and immunomodulatory effects by modulating inflammatory mediators and cytokine production (22, 23). BV has long been used in traditional East Asian medicine and continues to be widely used in the management of various musculoskeletal disorders, including osteoarthritis, gout, and RA, owing to these therapeutic properties (24–27). Although BV has been widely used in clinical practice, AEs, including allergic reactions, have been reported following its use (28, 29). Therefore, a comprehensive evaluation of its therapeutic efficacy and safety is essential to support its evidence-based clinical use.

The effectiveness and safety of BV have been systematically reviewed across various musculoskeletal disorders; however, evidence specific to RA remains limited (27). Although previous systematic reviews focusing on RA have been published (30–32), they included a relatively small number of randomized controlled trials (RCTs), limiting further analyses such as stratification by treatment effectiveness rate (TER) response criteria and subgroup analyses according to concomitant medications. Therefore, we aimed to provide an updated and superiorly comprehensive synthesis of the available evidence by incorporating recently published RCTs, performing sensitivity analyses using a consistent TER cutoff, conducting subgroup analyses according to concomitant medications, and evaluating effectiveness and safety outcomes to provide a superiorly robust assessment of the clinical evidence.

## 2 Materials and methods

### 2.1 Protocol and registration

This systematic review was conducted in accordance with the Preferred Reporting Items for Systematic Reviews and Meta-Analyses (PRISMA) 2020 statement (Supplementary Table S1) (33). The protocol of this review was registered in International Prospective Register of Systematic Reviews (PROSPERO; CRD42021238058) and subsequently published (34)

### 2.2 Search strategy

We conducted a comprehensive search of all databases on March 28, 2026, with no restriction on publication year, to identify RCTs evaluating BV for RA. Specifically, we searched the following electronic databases: PubMed, Embase, the Cochrane Library, China National Knowledge Infrastructure (CNKI), Citation Information by NII (CiNii), Scientific Electronic Library Online (SciELO), Korean Studies Information Service System (KISS), ScienceOn, and the Oriental Medicine Advanced Searching Integrated System (OASIS). Additionally, citation searching of previous systematic reviews was performed to identify potentially eligible studies. The search strategy was developed by combining Medical Subject Headings, Emtree terms, and free-text terms related to RA and BV (Supplementary Table S2).

### 2.3 Eligibility criteria

We included studies involving patients diagnosed with RA, regardless of race, age, or sex, according to either the 1987 American College of Rheumatology (ACR) or 2010 American College of Rheumatology/European Alliance of Associations for Rheumatology (ACR/EULAR) classification criteria. Studies that included patients diagnosed with other forms of arthritis, such as osteoarthritis, were excluded. Only interventions involving the injection of BV into treatment points were included, regardless of the injection technique, whereas other administration routes, such as oral and topical administration, were excluded. To evaluate the effects of BV, we included head-to-head comparison studies and add-on therapy studies in which the same co-intervention was administered to both groups. No restrictions were applied to comparator interventions, which included sham treatment and conventional medication. Eligible study designs were limited to RCTs. Observational, non-randomized, laboratory, and other non-clinical studies were excluded. No restrictions were placed on publication year, type or language. The primary outcomes included indicators of RA improvement, such as pain, disease activity–related outcomes, and TER. The secondary outcomes included RA-related laboratory markers, including erythrocyte sedimentation rate (ESR) and C-reactive protein (CRP), as well as the incidence of AEs.

### 2.4 Study selection and data extraction

Two reviewers (J.H. and W-S.S.) independently performed study selection and data extraction. Any disagreements were resolved through discussion, and unresolved disagreements were adjudicated by a third reviewer (J-H.K.). After duplicate records had been removed, the titles and abstracts were screened to exclude studies that did not meet the predefined eligibility criteria. The full texts of potentially eligible studies were then assessed for final inclusion. Bibliographic records were managed using EndNote 21 (Clarivate Analytics). Data from the included studies were extracted using a prespecified Microsoft Excel data extraction form. When outcome data were available only in graphical format, the corresponding authors were contacted to request the numerical data. The following data were extracted: first author, publication year, participants’ mean age, sex, disease duration, sample size, details of the intervention and comparator, outcomes, and AEs.

### 2.5 Quality assessment

Two reviewers (J.H. and W-S.S.) independently assessed the risk of bias and certainty of evidence. Any disagreements were resolved through discussion, and unresolved disagreements were adjudicated by a third reviewer (J-H.K.). The risk of bias was assessed using the Cochrane Risk of Bias 2.0 (RoB 2.0) tool, which evaluates five domains: bias arising from the randomization process, deviations from intended interventions, missing outcome data, measurement of the outcome, and selection of the reported result (35). Each domain was rated as “low risk,” “some concerns,” or “high risk.” The certainty of evidence for each outcome was assessed using the Grading of Recommendations, Assessment, Development, and Evaluation (GRADE) approach. The certainty of evidence was rated as high, moderate, low, or very low based on five domains: risk of bias, inconsistency, indirectness, imprecision, and publication bias.

### 2.6 Statistical analysis

Meta-analyses were performed using the Review Manager software (version 5.4; The Nordic Cochrane Center, The Cochrane Collaboration, Copenhagen, Denmark). Dichotomous outcomes were analyzed using risk ratios (RRs) with the Mantel–Haenszel method under a random-effects model. Continuous outcomes were analyzed using mean differences (MDs) with the inverse-variance method under a random-effects model when the same measurement scales were used, whereas standardized mean differences (SMDs) were applied when different scales were used to assess the same outcome. All effect estimates were reported with 95% confidence intervals (CIs) and presented as forest plots. Statistical heterogeneity was assessed using the Chi-square test and Higgins I^2^ statistic with the following interpretations: 0%–40%, unimportant heterogeneity; 30%–60%, moderate heterogeneity; 50%–90%, substantial heterogeneity; and 75%–100%, considerable heterogeneity (36). Additionally, subgroup analyses were performed to examine the effects of different intervention methods and explore potential sources of heterogeneity. For meta-analyses including at least 10 studies, potential publication bias was evaluated using Egger's regression test, and funnel plots were generated for visual inspection of asymmetry.

## 3 Results

### 3.1 Search results

A total of 854 records were identified through database searching. After the removal of 134 duplicate records, 720 records underwent title and abstract screening. Of these, 72 studies were considered eligible for full-text assessment. An additional six studies were identified through citation searching, resulting in a total of 78 full-text articles assessed for eligibility. Ultimately, 17 studies (37–53) met the inclusion criteria, of which 14 were included in the meta-analysis. The remaining three studies were excluded from the quantitative synthesis owing to a co-intervention (37), an unclear intervention duration (38), and the use of a sham comparator (39), respectively. Among the included studies, 13 (40–52) compared BV with Western medicine (WM), including DMARDs, whereas two studies (52, 53) compared BV with electroacupuncture (EA). One study contributed to both comparisons (Figure 1).

**Figure 1:**
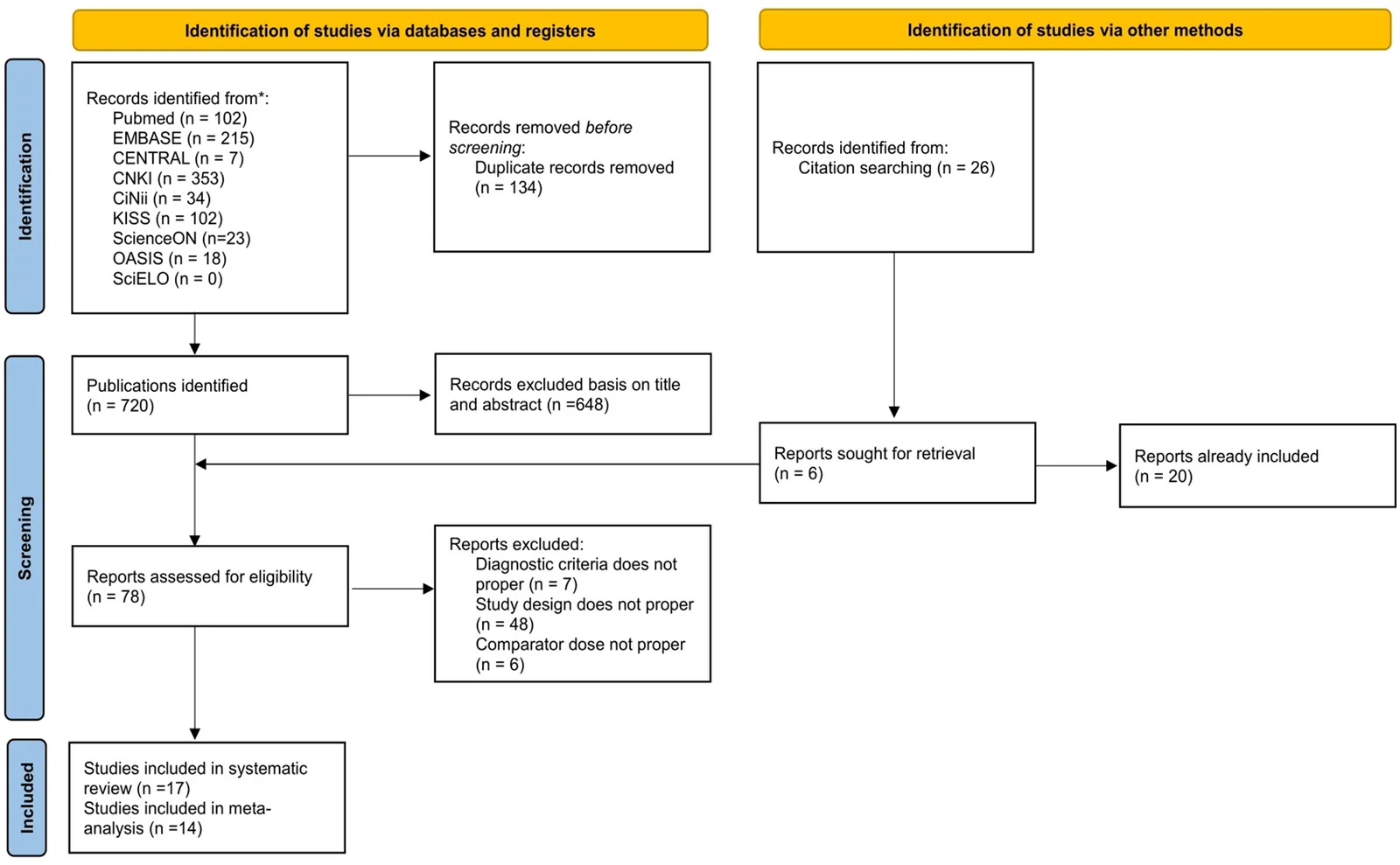
PRISMA flow diagram. CENTRAL, The Cochrane Central Register of Controlled Trials; EMBASE, Excerpta Medica database; CNKI, China National Knowledge Infrastructure; CiNii, Citation Information by NII; SciELO, Scientific Electronic Library Online; KISS, Korean Studies Information Service System; OASIS, Oriental Medicine Advanced Searching Integrated System.

### 3.2 Characteristics of the included studies

The 17 included RCTs enrolled 1,304 patients with RA diagnosed according to either the 1987 ACR or 2010 ACR/EULAR classification criteria. Of these, 642 patients were allocated to the BV group, 602 to the pharmacological treatment group, and 60 to the EA group. Regarding the type of BV intervention, one study (39), conducted in Korea, used diluted purified BV injections, whereas the remaining 16 studies (37, 38, 40–53), all conducted in China, used live bee stings. Among the 16 studies comparing BV with WM, 15 used csDMARDs, all of which included MTX. Of these, three studies combined MTX with SSZ, and one combined MTX with LEF. In addition to csDMARDs, concomitant medications included corticosteroids or nonsteroidal anti-inflammatory drugs (NSAIDs), such as diclofenac, nabumetone, celecoxib, and meloxicam, whereas one study used NSAIDs alone. The mean disease duration varied widely across studies, ranging from several months to more than 15 years. Most studies included patients with established RA, whereas four studies (40, 44, 48, 49) enrolled patients with relatively early disease, with a mean disease duration of approximately 1–2 years (Table 1).

**Table 1:** Characteristics of the included studies.

First Author (Year)	Patient		Intervention			Treatment duration	Outcome
	(Male/female)	Duration (mean ± SD)	Intervention Group(I)	Control Group(C)	Control Group2(C2)		
(39)	I: 37 (4/33) C: 32 (4/28)	I: 7.3 ± 4.6 years C: 9.2 ± 7.0 years	BV - bee venom (NR) 1 mg/3,000cc diluted 0.2 mL/session (max 1.0 mL) - 2 sessions/week	Sham - Normal saline 0.2 mL/session (max 1.0 mL) 2 sessions/week	None	2months	1. Symptoms 2. Quality of life (HAQ, pain VAS) 3. Laboratory (ESR, CRP)
(46)	I: 30 (6/24) C: 30 (6/24)	12.34 ± 5.96 years	BV - bee-sting (NR), 15 min retention, 1 session/1–2days - Start with 1–2 bees, increased according to symptom	WM - MTX 10 mg, PO, once every week - Celecoxib 200 mg, PO, once daily	None	60days	1. TER 2. Laboratory (RF)
(50)	I: 60 (9/51) C: 60 (10/50)	I: 5.7 ± 3.2 years C: 5.2 ± 2.9 years	BV - bee-sting (*Apis cerana*), 15 min retention, 5–15 bees/session, 3sessions/week	WM - MTX 10 mg, PO, once every week - Celecoxib 200 mg, PO, once or twice daily (according to symptom)	None	8weeks	1. TER 2. Symptoms 3.Laboratory (RF, ACCP, ESR, CRP) 4. AEs
(44)	I: 30 (7/23) C: 30 (8/22)	I: 22.87 ± 6.3 months C: 25.37 ± 6.76 months	BV - bee-sting (*Apis cerana*), 15 min retention, every other day, 3sessions/week - Start with 2 bees/session, increased by 1–2 bees/session, average 10–20 bees/session	WM - MTX 10 mg, PO, once every week - Celecoxib 200 mg, PO, once daily	None	8weeks	1. TER 2. Symptoms 3. Laboratory (ESR) 4. DAS 28 5. AEs
(48)	I: 36 (21/15) C: 36 (23/13)	I: 12.45 ± 5.23 months C: 11.32 ± 4.16 months	BV - bee sting (NR), 5 min retention, every other day, 3sessions/week - Start with 1 bee, 6bees/session	WM - MTX PO 7.5 mg first week, 10 mg 2–8th week - Folate 0.8 mg PO, once every week, 2days after taking MTX	None	8weeks	1. TER 2. Symptoms 3, Laboratory (ESR, RF)
(45)	I: 30 (20/10) C: 30 (19/11)	I: 3.50 ± 0.86 years C: 3.42 ± 0.83 years	BV - bee sting (NR), 10 min retention, 2sessions/week, - increased by 1–2 bees/session, maximum 10bees	WM - MTX PO, 10 mg fist week, 15 mg 2–8th week	None	4weeks	1. TER 2. Symptoms 3. TCM Symptoms
(49)	I: 49 (13/36) C: 49 (15/34)	I: 1.08 ± 0.30 year C: 1.13 ± 0.39 year	BV - bee-sting (NR), 10 min retention, every other day, 3 sessions/week - Start with 1 bee, increased by 1 bee/session	WM - MTX: initial 7.5 mg, PO, once every week; increased by 2.5 mg/week to 15 mg - LEF 10 mg, PO, once daily - Diclofenac sodium SR 75 mg, PO as needed	None	8weeks	1. TER 2. AEs
(43)	I: 20 (8/12) C: 20 (9/11)	I: 0.5∼17 years C: 0.5∼19 years	BV - bee-sting (*Apis cerana*), 10–20 min retention, every other day, 3 sessions/week - Start with 1 bee, increased by 1 bee/acupoint, average 4–8 bees/acupoint	WM - MTX or AUR and NSAIDs - details are not reported	None	2months	1. TER 2. Symptoms 3. Laboratory (ESR, CRP, RF) 4. AEs
(53)	I: 30 (10/20) C: 30 (10/20)	I: 12.35 ± 8.68 years C: 11.53 ± 6.87 years	BV - bee-sting (NR), every other day, 3 sessions/week - Start with 1 bee, increased by 1–2 bee/session, average 5–15 bee/session	WM - Ibuprofen SR 1–2 capsules, PO, twice daily	EA - Number 30 needles, dense-disperse wave, 30 min/session, every other day, 3 sessions/week.	3months	1. TER 2. Symptoms 3. Laboratory (ESR, CRP, RF)
(52)	I: 40 (13/27) C: 30 (14/16)	I: 15.58 ± 5.24 years C: 13.95 ± 5.21 years C2: 16.58 ± 5.32 years	BV - bee-sting (NR), every other day, 3 sessions/week - Start with 1 bee, increased by 1–2 bee/session, average 5–10 bee/session - Nabumetone: as needed	WM - MTX: initial 5 mg, PO once every week; gradually increased to 10 mg - Folate 10 mg, PO, once every week (taken 24 h after MTX) - Nabumetone: as needed	EA - Number 30 needles, dense-disperse wave, 30 min/session, every other day, 3 sessions/week.	3months	1. TER 2. Symptoms 3. Laboratory (ESR, CRP, IL-1, IL-6, TNF-α, cortisol)
(41)	I: 20 (6/14) C: 20 (4/16) C2: 20 (4/16)	3 months∼20 years	BV + WM - bee-sting (NR), 5 min retention, every other day, 3 sessions/week - Start with 1 bee, increased by 2–3 bees/session - MTX 10 mg, PO, once every week	WM - MTX 10 mg, PO, once every week	WM - MTX 10 mg, PO, once every week - Prednisolone 7.5 mg, PO, once daily	2months	1. TER 2. Symptoms 3. Laboratory (RF, ESR, CRP) 4. AEs
(42)	I: 50 (7/43) C: 50 (5/45)	I: 5.03 ± 2.95 years C: 4.85 ± 2.55 years	BV + WM - bee-sting (NR), every other day - Start with 1 bee/session, increased by 1–2 bee/session, average 8–15 bees/session - WM: same as control group	WM - MTX 7.5 mg, PO, once every week - SSZ 0.5 g, PO, three times daily - MXM 7.5 mg, PO, twice daily	None	3months	1. TER 2. Symptoms 3. Laboratory (ESR, CRP, R, PLT, IgA, IgG, IgM, IL-1, IL-6, TNF-α) 4. AEs
(38)	I: 62 (14/48) C: 62 (12/50)	I: 1.4 ± 0.6 years C: 1.3 ± 0.5 years	BV + WM - bee-sting (*Apis cerana*), details are not reported - WM: Same as control group	WM - MTX 20 mg, PO, once every week	None	NR	1. TER 2. Laboratory (CRP, IL-1, IL-6, IgA, IgG, IgM, Complementary)
(47)	I: 25 (7/18) C: 25 (8/17) C2: 25 (7/18)	I: 2.92 ± 1.26 years C: 2.78 ± 4.23 years C2: 2.64 ± 1.99 years	BV + WM - bee-sting (NR), 5–15 min retention, every other day 3 sessions/week - Start with 1–2 bees, increased by 1–2 bees/session, average 5–15 bees/session. - WM: Same as control groups	WM - MTX: Initial 5 mg, increased by 2.5 mg/week to a maximum of 10 mg, once every week - Prednisolone: 7.5 mg, PO, once daily	BV - Same as experimental group	2months	1. TER 2. Symptoms 3. HAQ 4. Laboratory (ESR, CRP, RF)
(40)	I: 30 (8/22) C: 30 (9/21)	I: 3.92 ± 4.15 months C: 3.78 ± 4.31 months	BV + WM - bee-sting (*Apis mellifera ligustica*) every other day, 3 sessions/week - Start with 1 bee, increased by 1–2 bee/session, average 8–15 bees/session - WM: same as control group	WM - MTX: Initial 5 mg, increased by 2.5 mg/week to a maximum of 10 mg, PO, once every week - Prednisolone: 7.5 mg, PO, once daily	None	2months	1. TER 2. Symptoms
(51)	I: 45 (24/21) C: 45 (26/19)	I: 6.65 ± 1.65 years C: 6.74 ± 1.42 years	BV + WM - bee-sting (*Apis cerana*), every other day －Start with 1 bee/acupoint, increased by 1–2 bee/session, average 8–15 bees/acupoint every other day - WM: same as control group	WM - MTX 7.5 mg, PO, every week - SSZ 0.5 g, PO, three times daily - MXM 7.5 mg, PO, twice daily - Gradually reduce the dose as the patient's symptoms improve	None	3months	1. TER 2. Symptoms 3. AEs
(37)	I: 23 (6/17) C: 23 (5/18)	I: 3.6 years C: 3.4 years	BV + WM + drain - bee-sting (*Apis mellifera ligustica*), 2–3 sessions/week - Start with 1 bee, increased by 1–2 bees/session, average 2–15 bees/session - WM: same as control group	WM + drain - MTX 7.5 mg, PO, every week - SSZ 0.5 g, PO, three times daily - MXM 7.5 mg, PO, two times daily	None	3months	1. TER 2. Lequesene index, VAS, HSS 3. Sonography 4. Laboratory (ESR, CRP) 5. AEs

SD, standard deviation; BV, bee venom; ESR, erythrocyte sedimentation rate; CRP, C-reactive protein; WM, western medicine; MTX, methotrexate; PO, per os; TER, total effective rate; RF, rheumatoid factor; RR, risk ratio; ACCP, anti-cyclic citrullinated peptide antibody; DAS 28, disease activity score in 28 joint; AUR, auranofin; AE, adverse event; NSAIDs, non-steroidal anti-inflammatory drug; VAS, visual analog scale; EA, electro-acupuncture; IL, interleukin; TNF-α, tumor necrosis factor-α; Ig, immunoglobulin; SSZ, sulfasalazine; MXM, meloxicam; HAQ, health assessment questionnaire; HSS; hospital for special surgery knee score.

### 3.3 Purified BV versus sham BV

Only one study (39) compared the effects of purified BV with those of sham BV. In the BV group, 1 mg of dried BV powder was diluted in 3,000 cc of solution and administered using a stepwise dose-escalation protocol, beginning with 0.2 mL and increasing to a maximum dose of 1.0 mL. The sham group received an identical volume of normal saline. Most participants were receiving DMARDs, with no significant between-group differences in baseline medication use. Treatments were administered twice weekly for 2 months. The assessed outcomes included morning stiffness, the Korean Health Assessment Questionnaire score, pain measured using a visual analog scale (VAS), ESR, and CRP. After the 2-month treatment period, the BV group demonstrated significantly greater improvements than the sham BV group across all outcome measures (all *p* < 0.05).

### 3.4 Head-to-head comparison

#### 3.4.1 BV (bee sting) versus WM

##### 3.4.1.1 Clinical outcomes

Joint pain score (JPS) was assessed using various measurement scales across the included studies, with treatment effects generally favoring the BV group. Two studies evaluated pain using a VAS. One study reported a significantly greater reduction in VAS scores in the BV group (from 7.00 ± 1.74 to 4.28 ± 1.07) than in the WM group (from 7.30 ± 1.42 to 5.42 ± 1.10) (53). This between-group difference remained significant at the 3-month follow-up, with mean VAS scores of 3.23 ± 0.87 in the BV group and 4.75 ± 0.87 in the WM group. The other study also reported a significant reduction in VAS scores after BV treatment; however, detailed numerical data were not available (50). The meta-analysis showed that reductions in JPS and pain joint count (PJC) were significantly greater with BV than with WM (JPS: SMD −0.99, 95% CI −1.37 to −0.60; I^2^ = 20%; PJC: SMD −0.76, 95% CI −1.31 to −0.21; I^2^ = 69%). However, substantial heterogeneity was observed for PJC (Figure 2).

**Figure 2:**
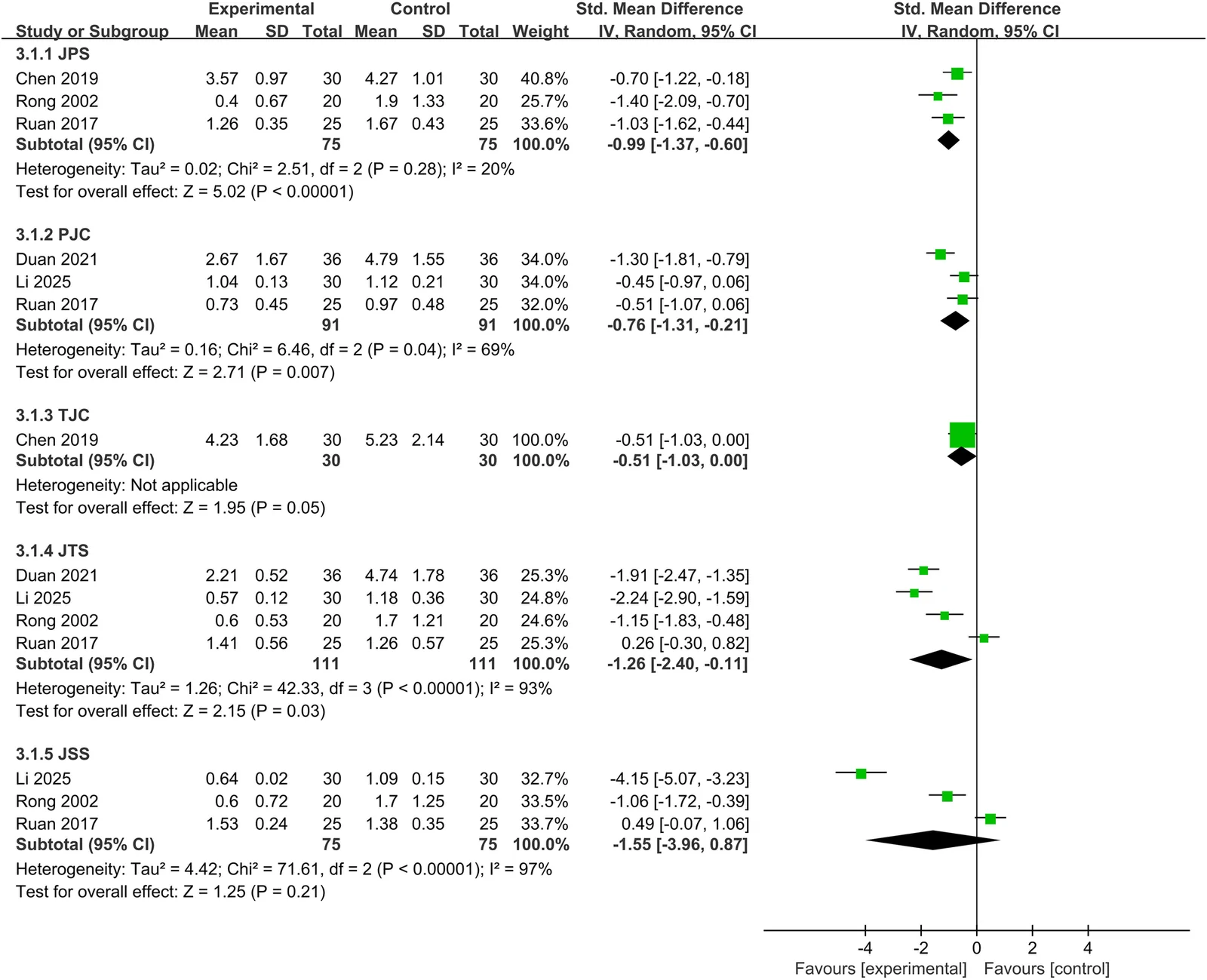
Forest plot of the standard mean difference for pain and disease activity-related symptoms in the head-to-head comparison of bee venom and western medicine.

The Disease Activity Score in 28 joints (DAS28) based on ESR, a composite measure of disease activity, was reported in only one study. This study showed a significantly greater improvement in the BV group (from 5.33 ± 0.28 to 3.86 ± 0.33) than in the WM group (from 5.33 ± 0.27 to 4.22 ± 0.28; *p* = 0.003) (44). Individual disease activity–related outcomes, including joint tenderness score (JTS), tender joint count (TJC), joint swelling score (JSS), and inflammatory markers (ESR and CRP), showed inconsistent results across studies. Meta-analysis indicated that BV was associated with significant reductions in JTS (SMD −1.26, 95% CI −2.40 to −0.11; I^2^ = 93%) and ESR (MD −5.11, 95% CI −9.42 to −0.81; I^2^ = 74%). In contrast, no statistically significant between-group differences were observed for TJC (SMD −0.51, 95% CI −1.03 to 0.00; *p* = 0.05), JSS (SMD −1.55, 95% CI −3.96 to 0.87; I^2^ = 97%), or CRP (MD 0.51, 95% CI −2.15 to 3.18; I^2^ = 0%). Considerable heterogeneity was observed for several outcomes, particularly JTS and JSS (Figures 2,3).

**Figure 3:**
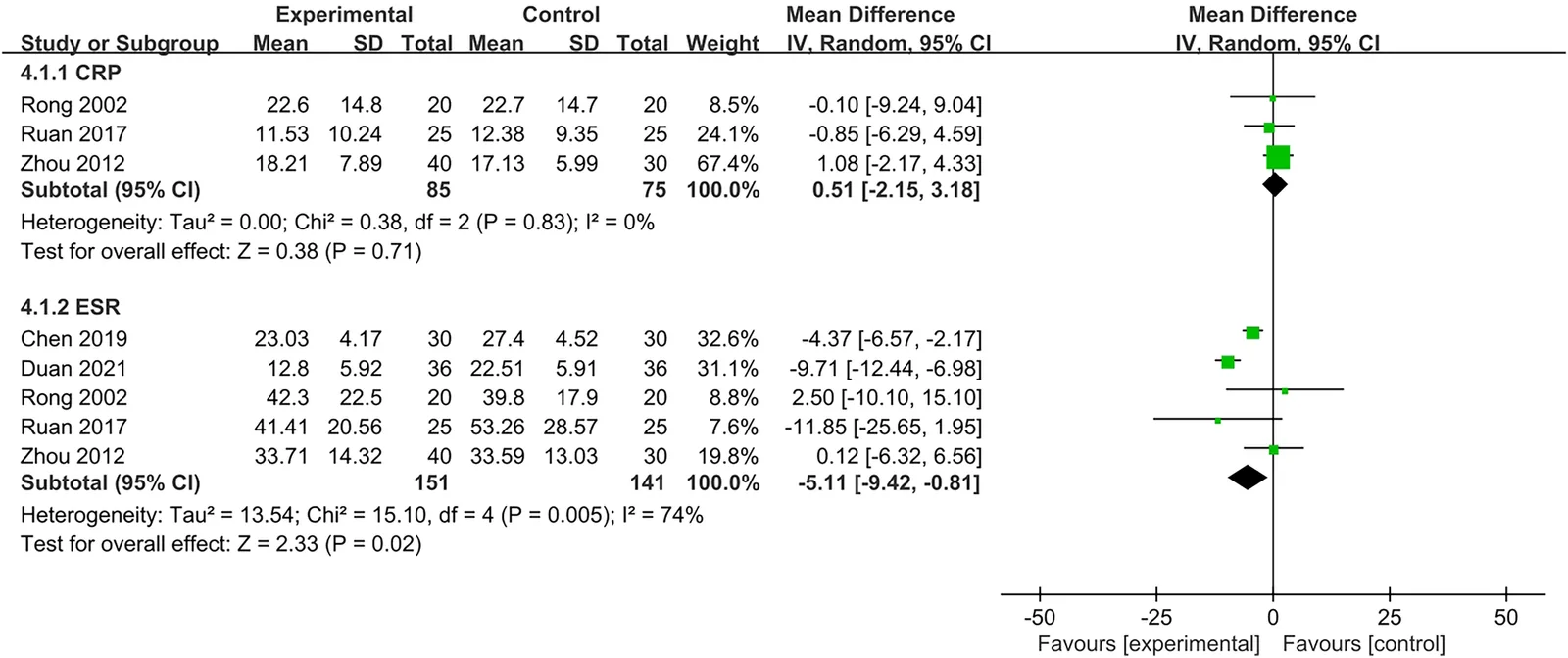
Forest plot of the mean difference for erythrocyte sedimentation rate and C-reactive protein in the head-to-head comparison of bee venom and western medicine.

Additional outcomes included the morning stiffness score (MSS) (SMD −1.29, 95% CI −2.71 to 0.13; I^2^ = 93%) and joint activity score (JAS) (SMD −0.38, 95% CI −1.28 to 0.53; I^2^ = 83%). Neither outcome showed a statistically significant difference between the BV and WM groups (Supplementary Figure S1).

##### 3.4.1.2 Treatment response

The TER was a composite outcome that categorized treatment response into four levels based on improvements in clinical symptoms and other study-specific outcome measures, including inflammatory markers. A sensitivity analysis was performed by including only studies that defined treatment response as a ≥ 30% improvement. Nine studies (43–50, 52) compared BV monotherapy with WM. The pooled analysis showed a slightly higher TER in the BV group than in the WM group (RR 1.07, 95% CI 1.01 to 1.12; I^2^ = 0%). However, this effect was no longer statistically significant in the sensitivity analysis restricted to studies using a ≥ 30% improvement criterion (RR 1.07, 95% CI 1.00 to 1.16; I^2^ = 0%; *p* = 0.06) (Figure 4).

**Figure 4:**
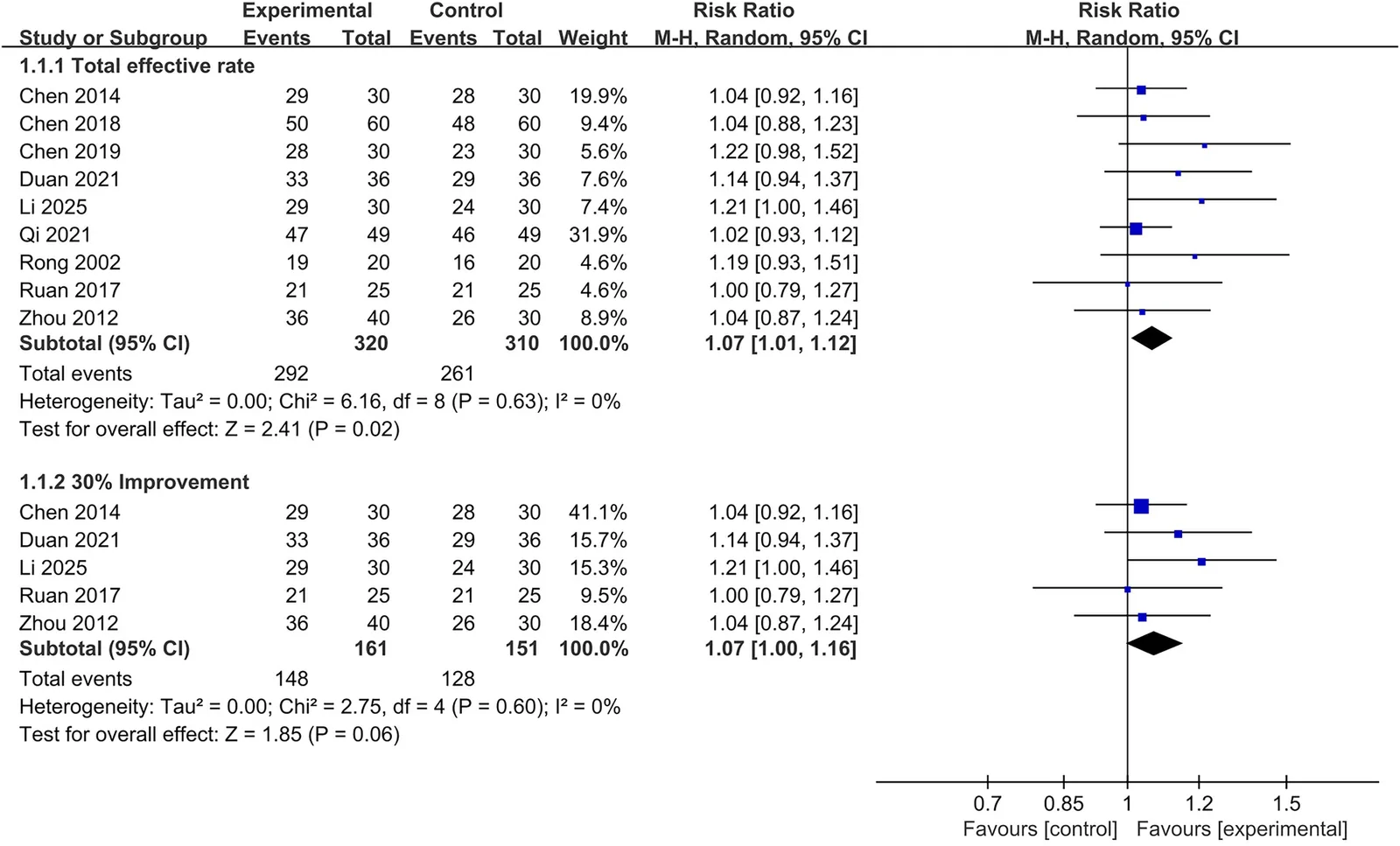
Forest plot of the risk ratio for total effective rate and 30% improvement rate in the head-to-head comparison of bee venom and western medicine.

Subgroup analyses according to the comparator medication regimen showed that the effect estimates for TER varied across medication regimens. The TER was significantly higher with BV than with MTX monotherapy (RR 1.12, 95% CI 1.01 to 1.25; I^2^ = 0%; *p* = 0.04). In contrast, no significant difference was observed when BV was compared with MTX combined with NSAIDs or corticosteroids (RR 1.07, 95% CI 0.99 to 1.16; I^2^ = 0%). One study compared BV with a combination of MTX, SSZ, and NSAIDs and found no significant difference in TER (RR 1.02, 95% CI 0.93 to 1.12). However, the test for subgroup differences was not significant (*p* = 0.43), indicating no evidence that the treatment effect of BV differed according to the comparator medication regimen (Supplementary Figure S2).

#### 3.4.2 BV (bee sting) versus EA

##### 3.4.2.1 Clinical outcomes

Two studies (52, 53) compared BV with EA as monotherapy. For pain outcomes, one study reported no significant between-group differences in pain measured using a VAS, either immediately after treatment or at the 3-month follow-up. VAS scores decreased from 7.00 ± 1.74 to 4.28 ± 1.07 in the BV group and from 6.98 ± 1.55 to 4.42 ± 1.01 in the EA group, with corresponding 3-month follow-up scores of 3.23 ± 0.87 and 3.98 ± 1.03, respectively. However, the same study reported a significantly greater reduction in JPS in the BV group than in the EA group (53).

Regarding disease activity–related outcomes, one study reported significant reductions in JTS and JAS in the BV group, whereas no significant between-group difference was observed for JSS (53). A meta-analysis of the two studies showed that the reductions in CRP (MD −10.46, 95% CI −20.21 to −0.72; I^2^ = 91%) and ESR (MD −17.54, 95% CI −22.63 to −12.46; I^2^ = 0%) were significantly greater with BV than with EA (Supplementary Figure S3).

##### 3.4.2.2 Treatment response

Both studies defined the TER as a ≥ 30% improvement. Meta-analysis showed no significant difference in TER between the BV and EA groups (RR 1.10, 95% CI 0.93 to 1.32; I^2^ = 39%) (Supplementary Figure S4).

### 3.5 BV (bee sting) as an add-on to WM

#### 3.5.1 Clinical outcomes

Only one study reported pain measured using a VAS score, demonstrating a significantly greater reduction in the BV group (from 6.79 ± 1.22 to 3.40 ± 1.45) than in the WM group (6.56 ± 1.41 to 4.17 ± 1.83) (37). This study was excluded from the meta-analysis because joint aspiration was performed in both groups in addition to the assigned interventions, resulting in a co-intervention that differed from that of the other included studies. The meta-analysis demonstrated that compared with WM alone, BV as an add-on therapy was associated with significantly greater reductions in JPS (SMD −1.14, 95% CI −2.11 to −0.17; I^2^ = 93%) and PJC (SMD −0.80, 95% CI −1.42 to −0.17; I^2^ = 61%). However, considerable heterogeneity was observed for both outcomes (Figure 5).

**Figure 5:**
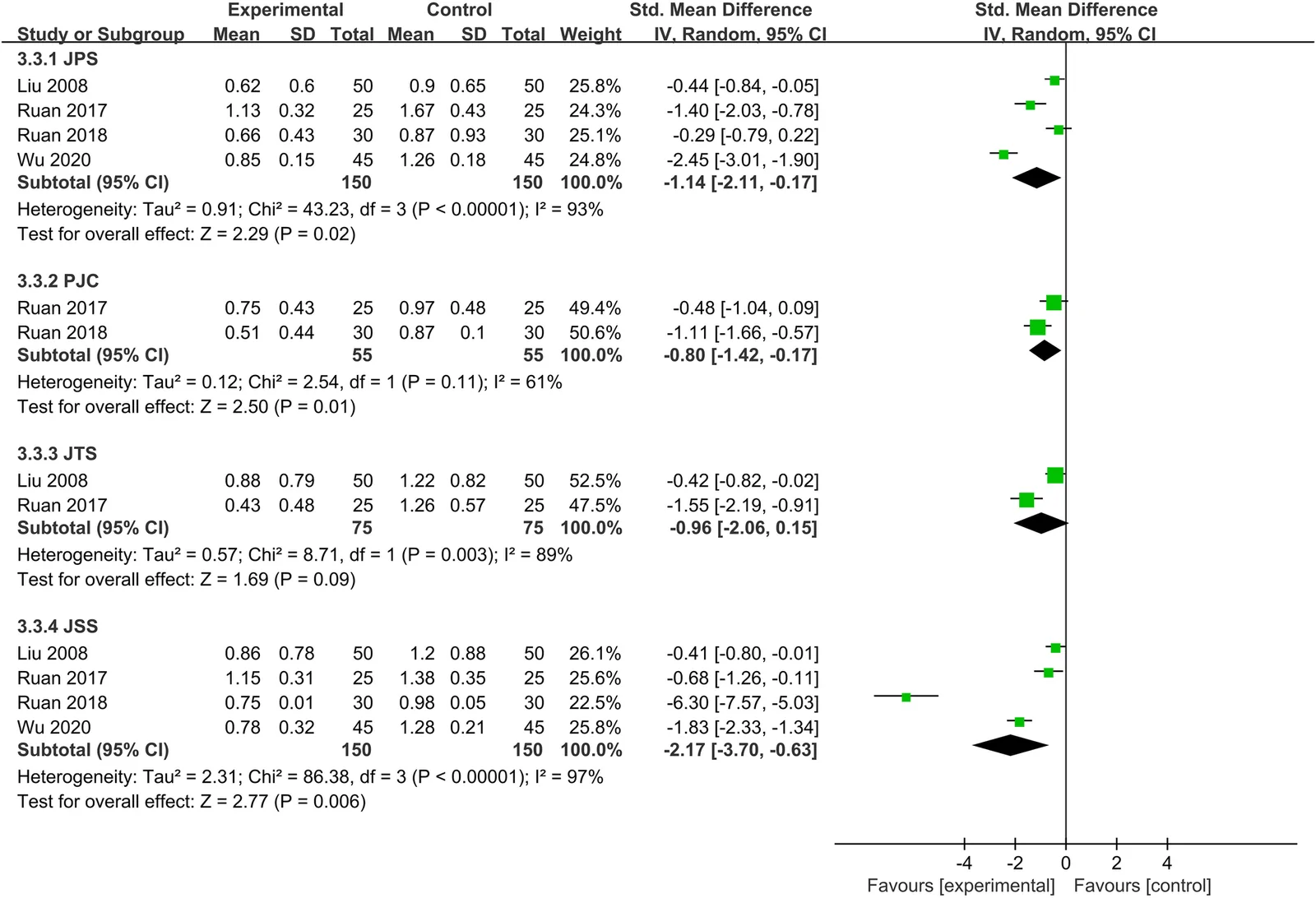
Forest plot of the standard mean difference for pain and disease activity-related symptoms in bee venom add-on therapy to western medicine.

No studies reported a composite measure of disease activity, such as the DAS28; the findings for individual disease activity–related outcomes were inconsistent. No significant between-group differences were observed for JTS (SMD −0.96, 95% CI −2.06 to 0.15; I^2^ = 89%) or CRP (MD −4.27, 95% CI −9.07 to 0.53; I^2^ = 73%). In contrast, BV was associated with significant reductions in JSS (SMD −2.17, 95% CI −3.70 to −0.63; I^2^ = 97%) and ESR (MD −9.88, 95% CI −19.24 to −0.52; I^2^ = 75%). Nevertheless, substantial heterogeneity was observed for these outcomes (Figures 5, 6).

**Figure 6:**
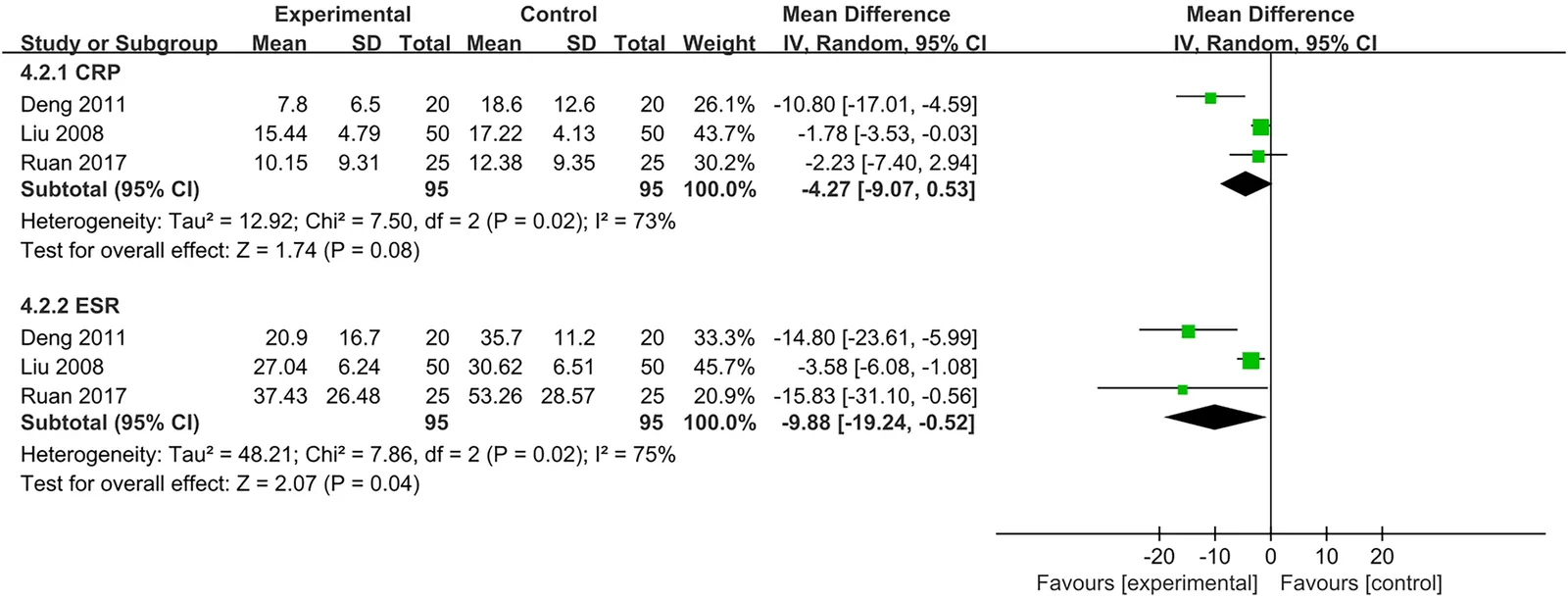
Forest plot of the mean difference for erythrocyte sedimentation rate and C-reactive protein in bee venom add-on therapy to western medicine.

Additional outcomes included the MSS and JAS. Neither MSS (SMD −0.78, 95% CI −1.69 to 0.12; I^2^ = 89%) nor JAS (SMD −1.41, 95% CI −3.22 to 0.40; I^2^ = 97%) showed a statistically significant difference between the BV and WM groups (Supplementary Figure S5).

#### 3.5.2 Treatment response

Five studies (40–42, 47, 51) compared BV as an add-on therapy with WM alone by evaluating the TER. The meta-analysis demonstrated a significantly higher TER in the BV add-on group than in the WM group (RR 1.20, 95% CI 1.10 to 1.32; I^2^ = 0%). In the sensitivity analysis restricted to studies defining TER as a ≥ 30% improvement, BV remained associated with a significantly high TER (RR 1.18, 95% CI 1.03 to 1.36; I^2^ = 9%) (Figure 7).

**Figure 7:**
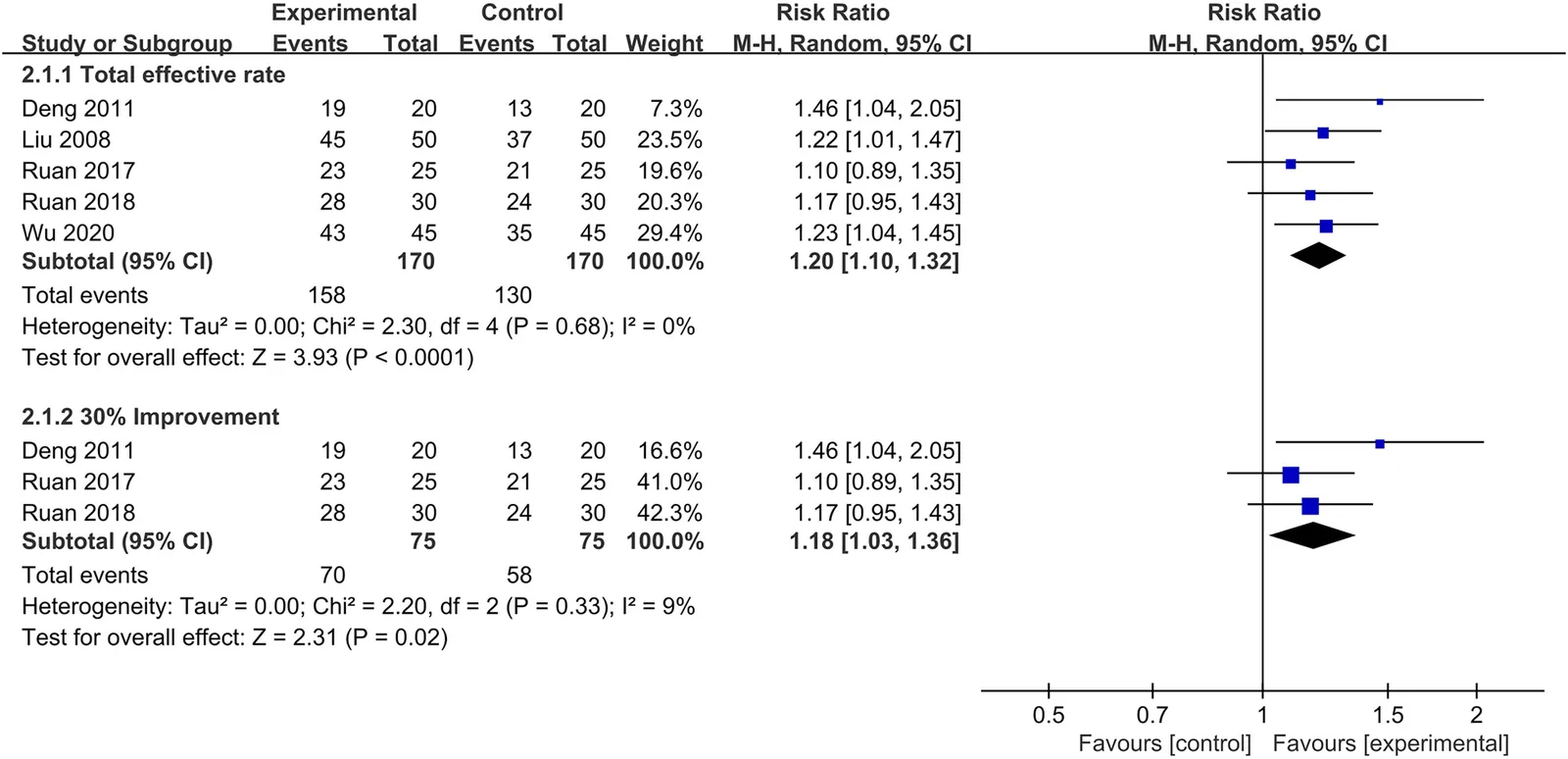
Forest plot of the risk ratio for total effective rate and 30% improvement rate in bee venom add-on therapy to western medicine.

Subgroup analyses according to the comparator medication regimen showed that the effect estimates for TER differed across treatment regimens. Compared with MTX monotherapy, BV was associated with a significantly higher TER (RR 1.46, 95% CI 1.04 to 2.05; *p* = 0.03), whereas no significant difference was observed when BV was compared with MTX combined with corticosteroids (RR 1.13, 95% CI 0.98 to 1.31; I^2^ = 0%). Two studies comparing BV with a combination of MTX, SSZ, and NSAIDs also showed a significantly higher TER in the BV group (RR 1.22, 95% CI 1.08 to 1.39; I^2^ = 0%) (Supplementary Figure S6).

### 3.6 AEs

Among the nine RCTs that reported AEs, five studies (37, 42, 43, 49, 51) provided data on the total number of patients who experienced at least one AE; the studies were included in the meta-analysis. In the head-to-head comparison, the risk of AEs was lower in the BV group (5/69, 7.2%) than in the WM group (17/69, 24.6%) (risk difference [RD] −0.17, 95% CI −0.34 to −0.01; I^2^ = 37%). Similarly, in the add-on therapy comparison, the risk of AEs was lower in the BV add-on group (16/118, 13.6%) than in the WM group (34/118, 28.8%) (RD −0.16, 95% CI −0.26 to −0.06; I^2^ = 0%) (Figure 8).

**Figure 8:**
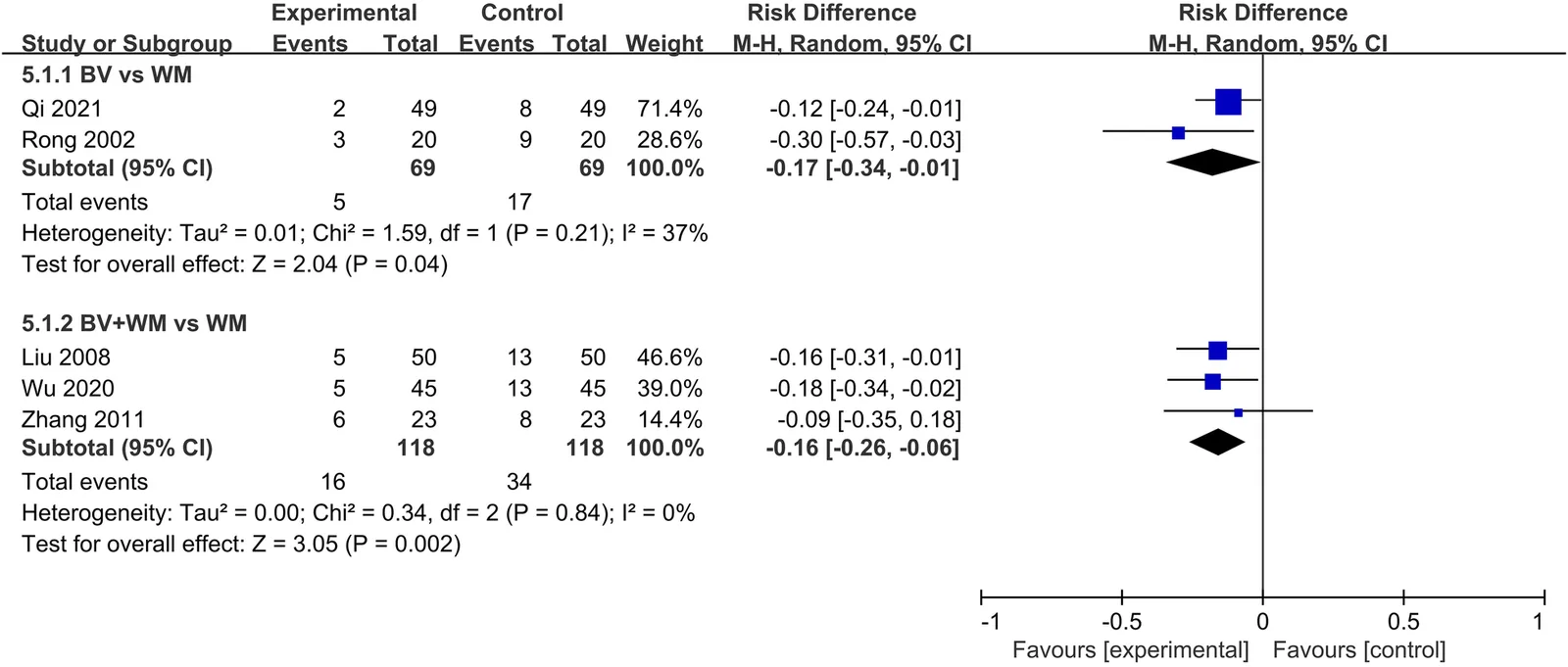
Forest plot of the risk difference for adverse events.

Among studies evaluating BV monotherapy (43, 44, 49, 50, 52), the most frequently reported AEs were itching (65 cases), followed by pigmentation (44 cases), swelling (44 cases), fever (14 cases), redness (eight cases), lymph node enlargement (three cases), urticaria (one case), and allergic reaction (one case). Two studies (44, 50)provided detailed descriptions of AEs and reported that events were mild and resolved within several days, whereas the remaining three studies did not report AE severity or recovery status. Serious adverse events (SAEs) were reported in three studies (43, 44, 50); no SAEs, including anaphylaxis, were reported in the BV groups. Details of the AE profiles are presented in Table 2.

**Table 2:** Safety profiles reported included studies.

Comparison	Study	Adverse events					
		Type of AE (cases)	No. of patient with AE	SAEs	Causality	Severity	Recovery
BV vs. Sham	(39)	Two participants dropped out due to AEs such as itching, but it was not reported which group they dropped out from.	NR	NR	NR	NR	NR
BV vs. WM	(50)	BV group: Itching (60), Pigmentation (44), Swelling (41), Low grade fever (9), Lymph node enlargement (2), Leukopenia (0), Urticaria (1) WM group: Itching (4), Pigmentation (7), Swelling (0), Low grade fever (1), Lymph node enlargement (0), Leukopenia (3), Urticaria (0)	NR	No serious adverse events reported including anaphylaxis.	NR	Mild	Self-limited.
	(44)	BV group: Itching and Redness (5), Low grade fever (2) WM group: No AE reported	NR	No serious adverse events reported including anaphylaxis.	NR	Mild	Self-limited. Within several days.
	(49)	BV group: Lymph node enlargement (1), Gastrointestinal AE (1) WM group: Dizziness (3), Lymph node enlargement (1), Gastrointestinal AE (4)	BV group (2/49) WM group (8/49)	NR	NR	NR	NR
	(43)	BV group: Fever, Redness and Swelling (3) WM group: Gastrointestinal AEs (nausea, pain, diarrhea, abdominal distention, anorexia), Rash etc. (9)	BV group (3/20) WM group (9/20)	Two participants of WM group dropped out due to SAE. Details are not reported	NR	NR	NR
	(52)	BV group: No AE reported WM group: NR	BV group (0/40) WM group (NR)	NR	NR	NA	NA
BV + WM vs. WM	(41)	BV group: Allergic reaction (3), Nausea (2) WM group: Nausea (3), Leukopenia (1)	NR	NR	NR	NR	NR
	(42)	BV group: Allergic reaction (4), Fever (1) WM group: Nausea and Anorexia (6), Dizziness (4), Leukopenia (3)	BV group (5/50) WM group (13/50)	NR	NR	NR	All AEs recovered after treatment.
	(51)	BV group: Allergic reaction (3), Fever (1), Nausea (1) WM group: Allergic reaction (5), Fever (2), Nausea (3), Anorexia (1), Dizziness (2)	BV group (5/45) WM group (13/45)	NR	NR	NR	NR
	(37)	BV group: Allergic reaction (1), Fever (2), Rash (3) WM group: Leukopenia (3), Abdominal pain (4), Dizziness (1)	BV group (6/23) WM group (8/23)	NR	NR	NR	All AEs recovered after treatment.

BV, bee venom; WM, western medicine; AEs, adverse effects; NR, not reported; SAE, serious adverse event.

### 3.7 Risk of bias

Regarding the randomization process, only one study (44) was judged to be at low risk of bias as it used a random number table for sequence generation and sealed envelopes for allocation concealment, with no significant between-group differences in baseline characteristics. The remaining studies did not provide adequate information on allocation concealment and were therefore judged as having some concerns. Regarding deviations from the intended interventions, only one study (39), which used a placebo to blind participants, was judged to be at low risk of bias. The remaining studies were conducted as open-label trials and did not report a study protocol; consequently, they were judged as having some concerns. Regarding missing outcome data, only one study (44), which reported no participant dropouts, was judged to be at low risk of bias. The remaining studies either did not report whether participant dropouts occurred or failed to provide reasons for missing outcome data; they were therefore judged as having some concerns. For the measurement of the outcome, two open-label studies (45, 49) that assessed only subjective clinical outcomes were judged to be at high risk of bias because no information on outcome assessor blinding was provided. Regarding the selection of the reported result, all studies were judged as having some concerns because no prespecified study protocol or statistical analysis plan was available. Overall, two studies (45, 49) were judged to be at high risk of bias, whereas the remaining studies were judged as having some concerns (Supplementary Figures S7 and S8).

### 3.8 Certainty of evidence

According to the GRADE assessment, the certainty of evidence ranged from low to very low across all outcomes. The certainty of evidence for the TER was rated as low in both comparisons, whereas the certainty of evidence for JTS, JPS, PJC, ESR, and CRP ranged from low to very low (Supplementary Table S3).

### 3.9 Publication bias

Publication bias was not assessed using funnel plots because fewer than 10 studies were available for each outcome.

## 4 Discussion

BV pharmacopuncture has traditionally been used in East Asia in various forms, including live bee stings and isolated or purified BV. In bee sting, a live bee is placed at the designated acupoint to deliver BV directly through its sting, whereas purified BV pharmacopuncture involves injection of prepared BV solution. The present review aimed to encompass these various forms of BV pharmacopuncture; however, only one included study evaluated purified BV, whereas all other included studies evaluated bee sting. Consequently, all studies included in the meta-analyses evaluated bee sting. Pain and disease activity are key clinical domains in RA, reflecting the patient-perceived symptom burden and underlying inflammatory activity of the disease (54). In this systematic review, BV pharmacopuncture using bee sting suggested potential benefits for pain-related outcomes, including JPS and PJC, in head-to-head and add-on therapy comparisons; however, substantial heterogeneity was observed in some analyses. To explore potential sources of this heterogeneity, we compared the procedural characteristics and baseline characteristics of participants across the included studies. Regarding the injection method, all studies used bee stings, and the treatment frequency (2–3 sessions per week) and treatment duration (2–3 months) were generally similar across studies. In contrast, the baseline characteristics of participants varied considerably, with the mean disease duration ranging from approximately 4 months to 12 years. Information on baseline disease activity and previous medication usage history was also limited or unclear in several studies. Although these factors may have potentially contributed to the observed heterogeneity, further exploration was limited by insufficient reporting and the small number of studies.

For disease activity, validated composite measures such as the DAS28 are considered the preferred assessment because they integrate multiple clinical and laboratory parameters into a single measure of overall disease status (55). However, DAS28 was reported in only one study, which showed a significant between-group difference favoring BV over WM (44). In the absence of sufficient data on validated composite measures, individual outcomes, such as joint assessment scores and inflammatory markers, cannot adequately substitute for a comprehensive assessment of disease activity. Consistent with this limitation, the results for these individual outcomes were not uniformly favorable. Similarly, in the head-to-head comparison with EA, BV was associated with significant reductions in both inflammatory markers (CRP and ESR). However, this finding was based on only two studies and was not consistently supported by other individual measures of disease activity. Collectively, the available evidence remains insufficient to draw firm conclusions regarding the effect of BV on overall disease activity because of the limited use of validated composite measures and inconsistent findings across individual disease activity outcomes.

Regarding treatment response, the TER was higher with BV than with WM in the primary analyses of the head-to-head and add-on therapy comparisons, consistent with the findings of previous systematic reviews (30, 31). However, the sensitivity analysis of the head-to-head comparison, restricted to studies defining TER as a ≥ 30% improvement, yielded only borderline statistical significance. This finding suggests that further evidence is required to confirm this effect. In contrast, the favorable effect of BV as an add-on therapy remained statistically significant in the corresponding sensitivity analysis. Overall, these findings suggest that BV can improve treatment response; however, the evidence should be interpreted cautiously because of the methodological and clinical heterogeneity among the included studies.

Despite the heterogeneity observed across clinical outcomes, the therapeutic effects of BV are biologically plausible given the multifaceted anti-inflammatory and antinociceptive properties of its bioactive components (23, 56). A recent systematic review of murine studies reported that melittin ameliorated experimental arthritis through anti-inflammatory, anti-osteoclastogenic, and antinociceptive mechanisms (57). Consistent with these findings, preclinical studies have shown that melittin suppresses Toll-like receptor–mediated signaling, inhibits nuclear factor-*κ*B activation, and reduces the production of pro-inflammatory cytokines, including tumor necrosis factor-α, interleukin-1β, and interleukin-6 (58, 59). Melittin has also been reported to inhibit receptor activator of nuclear factor-*κ*B ligand signaling, thereby potentially attenuating osteoclast differentiation and subsequent joint destruction (60–62). Additionally, BV may modulate nociceptive pathways through peripheral and central mechanisms, including the regulation of neuroimmune interactions and activation of descending inhibitory pathways (63, 64). Although these mechanistic findings do not establish the clinical efficacy of BV, they provide biological plausibility for the therapeutic effects observed in the present review.

Beyond efficacy, the safety profile of BV is an important consideration for its clinical application. In the present review, most AEs reported in the BV groups were localized reactions, and the meta-analyses showed that BV monotherapy and BV as an add-on therapy were associated with a lower risk of AEs compared with the respective WM comparator groups. These findings are generally consistent with those of a previous systematic review, which found that AEs reported in RCTs were predominantly mild local reactions and that no SAEs were identified, although severe systemic reactions have been reported in some case reports and case series (29). Despite these findings, the severity and management of AEs were inadequately reported in most studies, limiting a comprehensive assessment of the safety profile of BV. Furthermore, although no SAEs, such as anaphylaxis, were reported, the relatively small sample sizes and inadequate safety reporting precluded definitive conclusions regarding the risk of rare but potentially severe systemic reactions. Moreover, the relatively low incidence of AEs observed in the BV groups may partly reflect reduced exposure to concomitant medications resulting from improved symptom control, as suggested by two included studies (42, 51). Given the potential for rare systemic hypersensitivity reactions associated with BV exposure, appropriate patient screening, including an assessment of hypersensitivity risk, and careful clinical monitoring remain warranted.

Although the present findings suggest that BV has potential as an adjunctive treatment for symptom management in RA, the available evidence remains insufficient to support definitive recommendations for its clinical use. This is primarily attributable to methodological shortcomings, including concerns about the risk of bias and limitations in study design and reporting, which contributed to the generally low to very low certainty of the evidence. Therefore, the current uncertainty surrounding the clinical effects of BV should be interpreted as reflecting the limitations of the available evidence rather than the absence of therapeutic potential.

This study has some limitations. First, standardized outcome measures were insufficiently reported across the included studies, with limited use of validated composite disease activity indices, such as the DAS28, and established treatment response criteria, such as the ACR 20/50/70 response criteria. Additionally, the TER, which was frequently used as an efficacy outcome, was defined inconsistently across studies. To address this issue, we performed a sensitivity analysis restricted to studies that defined treatment response using an explicit ≥ 30% improvement criterion. Second, clinical heterogeneity among the included studies may have influenced the interpretation of the findings, while insufficient reporting limited a more comprehensive exploration of its potential sources. Third, most studies had relatively short follow-up periods (2–3 months), limiting the evaluation of sustained clinical benefits, including remission and long-term disease control, as well as the detection of rare or delayed AEs. Fourth, the certainty of evidence was limited by methodological shortcomings and imprecision. Many included studies had concerns regarding risk of bias related to the randomization process, allocation concealment, and blinding. Finally, most included studies were conducted in China, which may limit the generalizability of the findings to other populations and healthcare settings.

In conclusion, this systematic review suggests that BV may be associated with some benefits in pain-related outcomes and treatment response when used as an adjunctive treatment for RA. However, substantial heterogeneity across some outcomes and the low or very low certainty of evidence limit firm conclusions regarding the clinical effects of BV in RA. Regarding safety, no evidence of an increased risk of AEs was observed with BV; however, incomplete reporting of AE severity and the lack of long-term safety data precluded a comprehensive safety assessment. Further high-quality RCTs incorporating standardized treatment protocols, validated outcome measures, extended follow-up periods, and systematic efficacy and safety assessments are warranted to clarify the therapeutic role of BV in the management of RA.

## Data Availability

The original contributions presented in the study are included in the article/Supplementary Material, further inquiries can be directed to the corresponding author.
