# Supplementary material for: Effectiveness and safety of bee venom pharmacopuncture for rheumatoid arthritis-related pain and disease activity: a systematic review and meta-analysis
Source: Front Med (Lausanne). 2026 Oct 14;13:1954842. doi: 10.3389/fmed.2026.1954842 (PMC13713728; doi:10.3389/fmed.2026.1954842)
Supplement: Supplementary file 1 [file Datasheet1.docx]

Supplementary Material

# 1 Supplementary Figures and Tables

## 1.1 Supplementary Tables

**Table S1. PRISMA Checklist**

| **Section and Topic** | **Item #** | **Checklist item** | **Location where item is reported** |
| --- | --- | --- | --- |
| **TITLE** | | |  |
| Title | 1 | Identify the report as a systematic review. | Title |
| **ABSTRACT** | | |  |
| Abstract | 2 | See the PRISMA 2020 for Abstracts checklist. | Abstract |
| **INTRODUCTION** | | |  |
| Rationale | 3 | Describe the rationale for the review in the context of existing knowledge. | Introduction |
| Objectives | 4 | Provide an explicit statement of the objective(s) or question(s) the review addresses. | Last paragraph of Introduction |
| **METHODS** | | |  |
| Eligibility criteria | 5 | Specify the inclusion and exclusion criteria for the review and how studies were grouped for the syntheses. | Materials and Methods 2.2 Eligibility criteria |
| Information sources | 6 | Specify all databases, registers, websites, organisations, reference lists and other sources searched or consulted to identify studies. Specify the date when each source was last searched or consulted. | Materials and Methods 2.1 Search strategy |
| Search strategy | 7 | Present the full search strategies for all databases, registers and websites, including any filters and limits used. | Materials and Methods 2.1 Search strategy |
| Selection process | 8 | Specify the methods used to decide whether a study met the inclusion criteria of the review, including how many reviewers screened each record and each report retrieved, whether they worked independently, and if applicable, details of automation tools used in the process. | Materials and Methods 2.3.1 Study selection |
| Data collection process | 9 | Specify the methods used to collect data from reports, including how many reviewers collected data from each report, whether they worked independently, any processes for obtaining or confirming data from study investigators, and if applicable, details of automation tools used in the process. | Materials and Methods 2.3.2 data extraction |
| Data items | 10a | List and define all outcomes for which data were sought. Specify whether all results that were compatible with each outcome domain in each study were sought (e.g. for all measures, time points, analyses), and if not, the methods used to decide which results to collect. | Materials and Methods 2.3.2 data extraction |
|  | 10b | List and define all other variables for which data were sought (e.g. participant and intervention characteristics, funding sources). Describe any assumptions made about any missing or unclear information. | Materials and Methods 2.3.2 data extraction |
| Study risk of bias assessment | 11 | Specify the methods used to assess risk of bias in the included studies, including details of the tool(s) used, how many reviewers assessed each study and whether they worked independently, and if applicable, details of automation tools used in the process. | Materials and Methods 2.3.3 Risk of bias assessment |
| Effect measures | 12 | Specify for each outcome the effect measure(s) (e.g. risk ratio, mean difference) used in the synthesis or presentation of results. | Materials and Methods 2.4 Statistical analysis |
| Synthesis methods | 13a | Describe the processes used to decide which studies were eligible for each synthesis (e.g. tabulating the study intervention characteristics and comparing against the planned groups for each synthesis (item #5)). | Materials and Methods 2.4 Statistical analysis |
|  | 13b | Describe any methods required to prepare the data for presentation or synthesis, such as handling of missing summary statistics, or data conversions. | Materials and Methods 2.4 Statistical analysis |
|  | 13c | Describe any methods used to tabulate or visually display results of individual studies and syntheses. | Materials and Methods 2.4 Statistical analysis |
|  | 13d | Describe any methods used to synthesize results and provide a rationale for the choice(s). If meta-analysis was performed, describe the model(s), method(s) to identify the presence and extent of statistical heterogeneity, and software package(s) used. | Materials and Methods 2.4 Statistical analysis |
|  | 13e | Describe any methods used to explore possible causes of heterogeneity among study results (e.g. subgroup analysis, meta-regression). | Materials and Methods 2.4 Statistical analysis |
|  | 13f | Describe any sensitivity analyses conducted to assess robustness of the synthesized results. | Materials and Methods 2.4 Statistical analysis |
| Reporting bias assessment | 14 | Describe any methods used to assess risk of bias due to missing results in a synthesis (arising from reporting biases). | Materials and Methods 2.4 Statistical analysis |
| Certainty assessment | 15 | Describe any methods used to assess certainty (or confidence) in the body of evidence for an outcome. | Materials and Methods 2.5 Quality of evidence assessment |
| **RESULTS** | | |  |
| Study selection | 16a | Describe the results of the search and selection process, from the number of records identified in the search to the number of studies included in the review, ideally using a flow diagram. | Results 3.1 search ruslts |
|  | 16b | Cite studies that might appear to meet the inclusion criteria, but which were excluded, and explain why they were excluded. | Results 3.1 search ruslts |
| Study characteristics | 17 | Cite each included study and present its characteristics. | Results 3.2 Characteristics of included studies |
| Risk of bias in studies | 18 | Present assessments of risk of bias for each included study. | Results 3.7 Risk of bias |
| Results of individual studies | 19 | For all outcomes, present, for each study: (a) summary statistics for each group (where appropriate) and (b) an effect estimate and its precision (e.g. confidence/credible interval), ideally using structured tables or plots. | Table 1 |
| Results of syntheses | 20a | For each synthesis, briefly summarise the characteristics and risk of bias among contributing studies. | Results 3..4 Head to Head comparison and 3.5 Add-on therapy comparison |
|  | 20b | Present results of all statistical syntheses conducted. If meta-analysis was done, present for each the summary estimate and its precision (e.g. confidence/credible interval) and measures of statistical heterogeneity. If comparing groups, describe the direction of the effect. | Results 3..4 Head to Head comparison and 3.5 Add-on therapy comparison |
|  | 20c | Present results of all investigations of possible causes of heterogeneity among study results. | Results 3..4 Head to Head comparison and 3.5 Add-on therapy comparison |
|  | 20d | Present results of all sensitivity analyses conducted to assess the robustness of the synthesized results. | Results 3..4 Head to Head comparison and 3.5 Add-on therapy comparison |
| Reporting biases | 21 | Present assessments of risk of bias due to missing results (arising from reporting biases) for each synthesis assessed. | 3.9 Publication bias |
| Certainty of evidence | 22 | Present assessments of certainty (or confidence) in the body of evidence for each outcome assessed. | 3.8 Quality of evidence and Table 3 |
| **DISCUSSION** | | |  |
| Discussion | 23a | Provide a general interpretation of the results in the context of other evidence. | 2^nd^ and 3^rd^ paragraph of Discussion |
|  | 23b | Discuss any limitations of the evidence included in the review. | 2^nd^ and 3^rd^ paragraph of Discussion |
|  | 23c | Discuss any limitations of the review processes used. | last paragraph of Discussion |
|  | 23d | Discuss implications of the results for practice, policy, and future research. | last paragraph of Discussion |
| **OTHER INFORMATION** | | |  |
| Registration and protocol | 24a | Provide registration information for the review, including register name and registration number, or state that the review was not registered. | First paragraph of Materials and Methods |
|  | 24b | Indicate where the review protocol can be accessed, or state that a protocol was not prepared. | First paragraph of Materials and Methods |
|  | 24c | Describe and explain any amendments to information provided at registration or in the protocol. | First paragraph of Materials and Methods |
| Support | 25 | Describe sources of financial or non-financial support for the review, and the role of the funders or sponsors in the review. | Funding |
| Competing interests | 26 | Declare any competing interests of review authors. | Declaration of Competing Interest |
| Availability of data, code and other materials | 27 | Report which of the following are publicly available and where they can be found: template data collection forms; data extracted from included studies; data used for all analyses; analytic code; any other materials used in the review. |  |

**Table S2. Detailed Search strategy of PubMed**

|  | Searches Terms |
| --- | --- |
| #1 | “Arthritis, Rheumatoid” [Mesh] |
| #2 | “Arthritis, Rheumatoid” [TIAB] |
| #3 | #1 OR #2 |
| #4 | “Bee Venoms” [Mesh] |
| #5 | ((“Bee” [Title/Abstract]) AND (“venom” [Title/Abstract] OR “venoms” [Title/Abstract])) OR “Apitoxin” [Title/Abstract] OR “pharmacopuncture” [Title/Abstract] OR “pharmacoacupuncture” [Title/Abstract] OR “pharmaco-puncture” [Title/Abstract] |
| #6 | #4 OR #5 |
| #7 | #3 AND #6 |

Table S3. GRADE evidence profiles

| Comparison | Outcome | Number of RCTs | Number of participants | | Effect | certainty |
| --- | --- | --- | --- | --- | --- | --- |
|  |  |  | Experimental | Control |  |  |
| BV vs WM | TER | 9 | 292/320 | 261/310 | 59 more per 1,000 (from 8 more to 101 more) | ⨁⨁◯◯ Low^a,b^ |
|  | JTS | 4 | 111 | 111 | SMD 1.26 SD lower (2.4 lower to 0.11 lower) | ⨁◯◯◯ Very low^a,b,c^ |
|  | JPS | 3 | 75 | 75 | SMD 0.99 SD lower (1.37 lower to 0.6 lower) | ⨁⨁◯◯ Low^a,b^ |
|  | PJC | 3 | 91 | 91 | SMD 0.76 SD lower (1.31 lower to 0.21 lower) | ⨁◯◯◯ Very Low^a,b,c^ |
|  | ESR | 5 | 151 | 141 | MD 5.11 lower (9.42 lower to 0.81 lower) | ⨁◯◯◯ Very Low^a,b,c^ |
|  | CRP | 4 | 85 | 75 | MD 0.51 higher (2.15 lower to 3.18 higher) | ⨁⨁◯◯ Low^a,b^ |
| BV+WM vs WM | TER | 5 | 158/170 | 130/170 | 153 more per 1,000 (from 76 more to 245 more) | ⨁⨁◯◯ Low^a,b^ |
|  | JTS | 2 | 75 | 75 | SMD 0.96 SD lower (2.06 lower to 0.15 higher) | ⨁◯◯◯ Very Low^a,b,c^ |
|  | JPS | 4 | 150 | 150 | SMD 1.14 SD lower (2.11 lower to 0.17 lower) | ⨁◯◯◯ Very Low^a,b,c^ |
|  | PJC | 2 | 55 | 55 | SMD 0.8 SD lower (1.42 lower to 0.17 lower) | ⨁◯◯◯ Very Low^a,b,c^ |
|  | ESR | 3 | 95 | 95 | MD 9.88 lower (19.24 lower to 0.52 lower) | ⨁◯◯◯ Very Low^a,b,c^ |
|  | CRP | 3 | 95 | 95 | MD 4.27 lower (9.07 lower to 0.53 higher) | ⨁◯◯◯ Very Low^a,b,c^ |

Abbreviations: GRADE, Grading of recommendations, assessment, development, and evaluation method; RCTs, Randomized controlled trials; BV, Bee venom; WM, Western medicine; TER, Total effective rate; JTS, Joint tenderness score; SMD, Standard mean difference; SD, Standard deviation; JPS, Joint pain score; PJC, Pain joint count; ESR, Erythrocyte sedimentation rate; MD, Mean difference; CRP, C-reactive protein.

a. Most studies evaluated as ‘some concerns’ or ‘High risk’ in most domains.

b. The results are not statistically significant, or the sample size is small

c. Substantial heterogeneity reflected by widely varying effect estimates and limited overlap of confidence interval

## 1.2 Supplementary Figures

Figure S1. Forest Plot of Standardized Mean Difference for Other Symptom Scores in the Head-to-Head Comparison of Bee Venom and Western Medicine

Figure S2. Subgroup Analysis of Total Effective Rate According to Medication Type in the Head-to-Head Comparison of Bee Venom and Western Medicine.

Figure S3. Forest Plot of Mean Difference for Erythrocyte Sedimentation Rate and C-Reactive Protein in the Head-to-Head Comparison of Bee Venom and Electroacupuncture.

Figure S4. Forest Plot of Risk Ratio for 30% Improvement Rate in the Head-to-Head Comparison of Bee Venom and Electroacupuncture.

Figure S5. Forest Plot of Standardized Mean Difference for Other Symptom Scores in Bee Venom Add-on Therapy to Western Medicine

Figure S6. Subgroup Analysis of Total Effective Rate According to Medication Type in Bee Venom Add-on Therapy to Western Medicine.

Figure S7. Summary of Risk of Bias

Figure S8. Risk of Bias bar Graph
